# A Possible Flow Cytometry-Based Viability and Vitality Assessment Protocol for Pathogenic *Vibrio cholerae* O1 and O139 Postexposure to Simulated Gastric Fluid

**DOI:** 10.1155/2021/5551845

**Published:** 2021-06-08

**Authors:** Atheesha Singh, Tobias George Barnard

**Affiliations:** Water and Health Research Centre, University of Johannesburg, Doornfontein 2028, South Africa

## Abstract

During the intake of contaminated water, for diarrheal disease to occur, *Vibrio cholerae* must survive through the bactericidal digestive secretion of gastric fluid during passage through the stomach. Determining the viability of these bacteria is challenging, with the standard cultivation methods for viability being time-consuming and unable to culture cells that may still function accordingly. This study assessed the use of enzyme action and membrane integrity as alternatives for determining vitality and viability, respectively, in gastric acid-stressed pathogenic *Vibrio cholerae* O1 and O139, using fluorescent probes thiazole orange (TO) for viability based on membrane integrity, carboxyfluorescein diacetate (CFDA) with acetoxymethyl ester (AM) for vitality based on metabolic activity, and propidium iodide (PI) for cell death/damage due to loss of membrane integrity, with flow cytometry. Simulated gastric fluid-treated bacterial cells were labelled with blends of TO+PI and CFDA-AM+PI, and these stained cells were separated into heterologous populations based on their fluorescence signal. The gastric acid exposed cells presented with high green fluorescence signals after staining with the metabolic probe CFDA-AM, which indicated intact (live) cells due to being metabolically active, whereas when the same cells were stained with the DNA probe (TO), these appeared to be in a “stressed state” due to loss of membrane integrity. Damaged cells (dead cells) showed high red fluorescence levels after staining with PI probe. The use of flow cytometry with fluorescent probes is a favorable method for evaluating the vitality and viability of bacteria when cells are labelled with a combination of CFDA-AM+PI.

## 1. Introduction

Intestinal (enteric) pathogenic bacteria, especially *Vibrio cholerae* (*V. cholerae*), are present in water environments of low temperature and almost neutral pH. In contrast, when these pathogenic bacteria enter a warm-blooded host, through the consumption of contaminated food or water, they will encounter an unfavorable environment such as acidic gastric fluid in the stomach before they reach the intestine to cause an infection [1]. Once ingested, in order to survive and adapt to the stressful environment, the *V. cholerae* alters its morphology to accommodate the in vivo cellular requirements [2]. The detergent-like properties of gastric fluid components such as pepsin and hydrochloric acid are responsible for the bactericidal and bacterial injury effect of gastric fluid. The way *V. cholerae* reacts with gastric fluid determines whether they can survive. According to Wu [3], when microorganisms are subjected to gastric fluid stress (or sublethal injury), several morphological changes may occur where structural and operational constituents such as the cell wall, membrane integrity enzyme activity, and nucleic acid contents are damaged or affected. The effectiveness of low pH gastric fluid as a bactericidal agent has been quantified using the typical cultivation methods, which is correct under standard procedures. However, this method assumes that only bacterial colonies which are present are alive [1, 4, 5], thereby underestimating the possibility of any viable but nonculturable bacteria.

According to Wu [3], it is important to remember that not all bacterial cells in a population will experience a similar extent of stress or injury and that varying stress conditions can induce different types of injury to the cell. Thus, measuring viability (ability of a cell to carry out all required functions for its existence under certain conditions) is important for determining bacterial physiology after stressful situations [6]. Distinct features of microbial viability are proliferation energy and membrane integrity [7]. Replicating (growing) bacterial cells can proliferate by colony counting, whereas nonreplicative (injured or dead) cells cannot. Metabolically active cells are generally referred to as being vital cells, and these cells are determined by nucleic acid synthesis, fermentation rate, ATP content, enzyme activity, preservation of membrane potential, and pH gradients [8]. Bacteria can change their physiological status (dormant or latent state) due to changes in environmental conditions, and they may be revived when favorable conditions return, i.e., stimulated to revert to a metabolically active state [9]. Culture-free evaluation of bacterial viability is crucial when (a) the bacteria are not culturable, (b) the results are needed urgently, and (c) when the mode of action or target of certain processes is of interest [10]. Roussel et al. [11] confirmed these changes when bacterial cells are exposed to the simulated stomach environment.

Flow cytometry has been used by various authors to study the viability of *Vibrio* species, without the requisite for cultivation on agar plates. This has included studying the *V. parahaemolyticus* cells entering and being resuscitated from the “viable but nonculturable state (VBNC)” in environmental water [12], growth characteristics of *V. cholerae* O1 in water [13], and the change in *V. cholerae* O1 in response to chlorine [14]. Roussel et al. [11] reported that when working with cells exposed to a gastric environment that entered an intermediate viability state, they could only be distinguished using flow cytometry and not with conventional plate counts, qPCR, and propidium monoazide-qPCR [11].

Staining technology with fluorescent probes has steered the advancement of procedures for measuring cellular functions for the live and dead estimation of bacterial cells [6]. According to [15], in their investigation, they showed that “small molecule fluorescent probes” can be used as vital tools for the visual analysis and capturing of organically active species due to probes “high sensitivity, good selectivity, and noninvasiveness, as well as high spatial and temporal resolution”. Bacterial cells must be further investigated to distinguish between subpopulations of viable, vital, dormant, and injured cells. Fluorescent probe staining is largely centred on different cellular physiological characteristics, such as: propidium iodide for cytoplasmic membrane permeability [10, 16], cyanine dyes for membrane potential [12], carboxyfluorescein diacetate acetoxymethyl ester (CFDA-AM) for esterase enzyme activity [17], and thiazole orange or SYBR Green I for relative DNA and nucleic acid content [1, 10]. Fluorescence probes can differentiate between “live and dead” bacterial cells as well as between “vigorous, frail, and injured microorganisms” [18]. These “viability” measurements are enhanced when used in a blend with flow cytometry (FCM), a procedure proficient in the quick investigation of single, fluorescent particles. With FCM, several factors of numerous fluorescently marked cells can be calculated in a few minutes. The cellular particles that “flow” through a fixed beam of light in a liquid stream are calculated in a flow cytometer by fluorescence signals and light scatter. The precise and instantaneous measurement of cellular particles by flow cytometry using fluorescent probes therefore permits for the detection of viable, vital, and intact bacteria cells that may or may not maintain membrane integrity [19]. FCM provides information on the heterogeneity of microbial subpopulations, which cannot be achieved by standard plate counts or biochemistry.

In a previous small-scale exploratory study [1], we showed that pathogenic nonacid adapted *V. cholerae* strains did have the capability to endure the simulated gastric acid fluid at low pHs in an “injured state” (due to the leaky membrane) which is presumably the VBNC state for survival. In this study, flow cytometry was able to show that non-acid-adapted *Vibrio cholerae* O1 and O139 are still metabolically active after exposure to simulated gastric fluid. The vitality (metabolic state) and viability (membrane integrity) of bacterial populations were examined by linking flow cytometric analysis with fluorescence staining. The collective use of propidium iodide (PI), carboxyfluorescein diacetate (CFDA) with acetoxymethyl ester (AM), and thiazole orange (TO) probes permitted the differentiation between viable and metabolically active bacterial cells, from bacterial cells with permeabilized or damaged cytoplasmic membranes (even when debris were in the samples).

## 2. Materials and Methods

### 2.1. *Vibrio cholerae* Exposure to Simulated Gastric Fluid (SGF)

**Figure 1:**
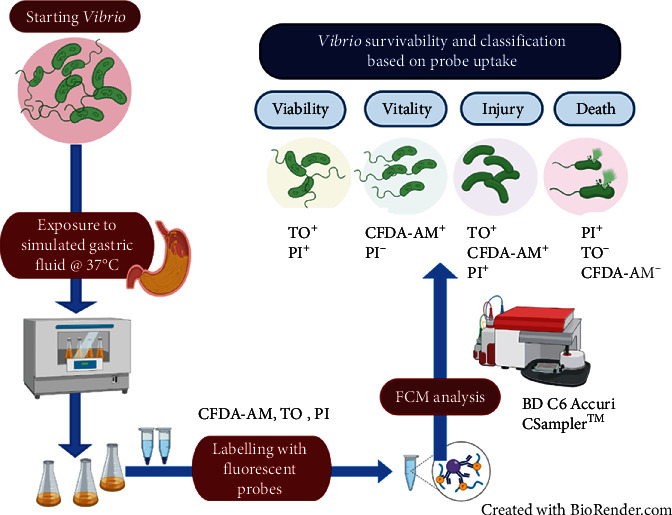
Exposure of *Vibrio* cells to simulated gastric fluid and classification after analysis.

The “National Health Laboratory Services” (NHLS) of South Africa supplied the *Vibrio cholerae* O1 (Ogawa) and *Vibrio cholerae* O139, from their National Stock Culture Collection. These bacteria were grown on culturable agar specific for *Vibrio* sp. to obtain pure colonies as described previously by Singh and Barnard [1]. From the pure colonies, the *V. cholerae* were then grown in nutrient broth (pH 7) (BioLab) to the late stationary phase to an optical density of ~0.9 OD_600nm_. Each batch of simulated gastric fluid (SGF) at pH 1.5, 2.5, 3.5, and 4.5 was prepared “fresh” before each experiment to ensure that bactericidal effects were not altered and stored at 4°C as published by Singh and Barnard [1] and as described by Yuk et al. [20] for up to 48 hrs. A total volume (*v*/*v*) of 98 ml of SGF medium was dispensed into individual 250 ml sterilized Erlenmeyer flasks and preheated to 37°C before the addition of a 2 ml non-acid-adapted bacterial strain with an inoculum size of 10^2^ CFU/ml (of approximately 14.5‐14.8 × 10^5^ CFU/ml). This was performed separately for each bacterial strain and at all SGF pHs. The SGF batch treatments were incubated at 37°C with slight agitation at 100 rpm for 180 min (to mimic the normal stomach movement). Samples were collected and analysed at 0, 30, 60, 120, and 180 min, respectively. Time at 0 min occurred immediately after bacterial inoculation into the SGF. After each sampling time interval, 2 ml of each SGF reaction was dispensed into 2.5 ml Eppendorf tubes and centrifuged for 10 min at 6000 x g in triplicate. A washing step was done to remove any residual SGF from the bacterial pellets. Thereafter, each cell pellet was investigated for viability, vitality, and culturability.  Figure 1 represents an overview of the *Vibrio* exposure to acid stress followed by labelling with fluorescent probes and subsequent analysis with flow cytometry (FCM) for bacterial cell survivability. Bacterial cell culturability after gastric fluid exposure was analysed as described in our previous study for the formation of “alive” bacterial cells [1].

### 2.2. Labelling of *Vibrio cholerae* Cells with Fluorescent Probes

*Vibrio cholerae* cell pellets and the corresponding controls were each labelled separately using the esterase metabolic probe “5-carboxyfluorescein diacetate with acetoxymethyl ester” (CFDA-AM [Invitrogen]), BD™ cell viability kit (BD Biosciences) comprising of thiazole orange (TO) and propidium iodide (PI) that only enters bacterial cells with damaged membranes (Figure 1). CFDA-AM (25 mM) is a cell permeable esterase substrate and was used in combination with (20 mM) propidium iodide (PI [Invitrogen]) to evaluate the vitality of bacteria based on metabolic activity. The BD™ cell viability kit determines bacterial membrane integrity and comprised of two probes: thiazole orange (TO) (42 *μ*mol/l): a cell-permeant DNA probe, and PI (4.3 mmol/l). Each acid-treated bacterial cell pellet was first reconstituted in 350 *μ*l “flow staining buffer (pH 7.02, PBS, 10 mmol/L ethylene diamine tetra-acetic acid (EDTA) (Merck), 0.01% Tween®20 (Sigma Aldrich))” [1]. For viability, each suspension at varying pH and time interval was labelled with 5 *μ*l of TO (420 nmol/l), vortexed, and allowed to stand for 15 min, in the dark, prior to the addition of 3 *μ*l PI (43 *μ*mol/l). For vitality, each suspension at varying pH and time interval was labelled with 2 *μ*l of CFDA-AM (10 *μ*M) and 1 *μ*l PI (30 *μ*M), vortexed gently, and incubated in the dark at 37°C for 30 min. The stained and unstained cells served as controls, to ensure proper gating was set and to allow for compensation on the flow cytometer [1]. The following bacterial controls were prepared with each probe: (a) TO stained live untreated cells, (b) CFDA-AM-stained live untreated cells, (c) PI-stained untreated dead cells, and blends of (d) CFDA-AM+PI and TO+PI stained with a mixture of untreated live and dead cells, and (e) CFDA-AM+PI and TO+PI-stained simulated gastric fluid treated cells. The dead *V. cholerae* bacterial cells was prepared according to [1] by killing actively growing cells with 80% iso-propanol (Saarchem) for 30 min, with confirmation of death confirmed with flow cytometry.

### 2.3. Flow Cytometric (FCM) Analysis of Fluorescently Labelled *Vibrio cholerae*

**Figure 2:**
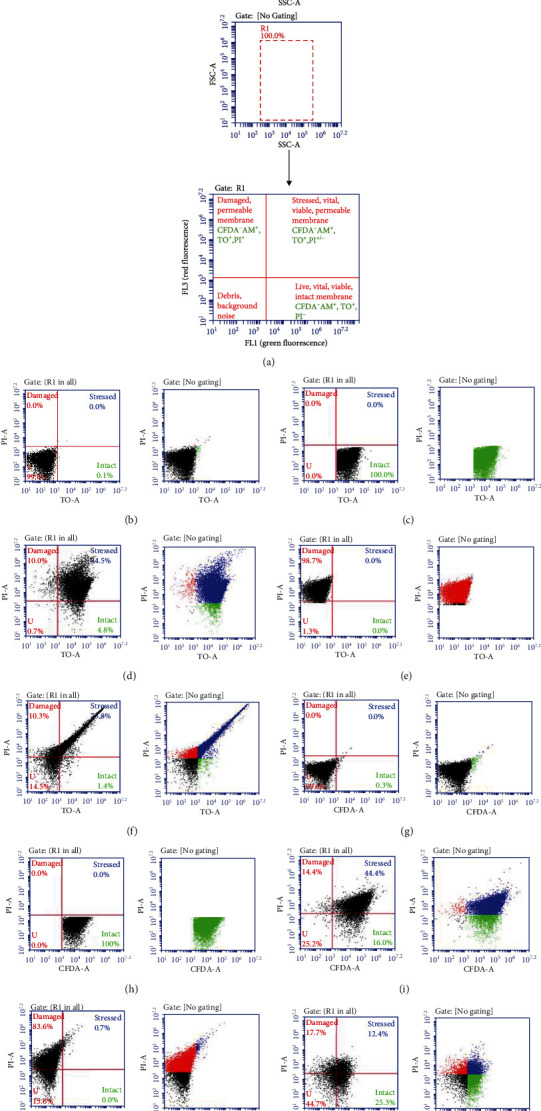
Flow cytometry multiparameter dot plots representing *Vibrio cholerae* O1 bacterial control cell suspensions stained with fluorescent probes: TO, CFDA-AM, and PI and analysed using a BD C6 Accuri flow cytometry system with CSampler™. The *x*-axis indicates the logarithmic green fluorescence intensity of CFDA-AM and TO (FL1). The *y*-axis indicates the logarithmic red fluorescence intensity of PI (FL3). Untreated bacterial controls include pure culture samples (b, c, d, g, h, and i) and dead control culture samples (d, e, i, and j). Treated simulated gastric fluid treated bacteria are represented on (f, k). Specific bacterial subpopulations are distinguished by scatter plots: gating of bacterial population on R1 (a) background noise/debris, unstained (black) cells (b, g) intact (green) population labelled with TO (c), or CFDA-AM (h). Mixed/stressed (blue) population labelled with TO+PI (d, f) or CFDA-AM+PI (i, k), and damaged (red) population labelled with PI (e, j).

Flow cytometry outlined the viability and vitality of *Vibrio cholerae* after labelling with fluorescent probes on the BD C6 Accuri flow cytometer with CSampler™ (Becton Dickinson (BD) Biosciences). Flow cytometer instrument quality control was performed immediately before each analysis using Spherotech 8- and 6-Peak Validation Beads (BD Biosciences) to ensure that the BD Accuri C6 was performing within the manufacturer's specifications. Data collection was performed using a 96 well U-bottom microtitre plate. Cell quantities were achieved on the flow cytometer by analyzing 100 *μ*l of each labelled sample at medium speed with a flow rate of 35 *μ*l/min, a core size of 16 *μ*m, with an acquisition threshold of 800 and an SSC-H threshold of 10000 to exclude unwanted particles. A wash step of two cycles according to the BD Accuri C6 instrument setup was performed after every sample run, to prevent cross contamination of samples, and the “instrument cleaning fluid cycle and extended clean of flow cell” was performed after each fluorescent probe was analysed to prevent any stain residue carryover. The BD Accuri flow cytometer machine comes with fluidized counting detectors that can count the number of constituents in a sample [10, 16]. The gating and labelling approach was used to confirm intact (viable)/damaged cell differences in all SGF exposed bacteria as illustrated in Figure 2. Fluorescence of TO and CFDA-AM occurred in the FL1 region with a “band-pass filter of 525 ± 25 nm,” and the fluorescence of PI was noted in the FL3 region with a “short-pass filter of 620 nm”. Fluorescence was observed using a “two-dimensional FL1-A (emission filter 533/30) vs. FL3-A (emission filter 670 LP)” on a logarithmic scale scatter plot. Compensation was performed to correct the overlay of one dye's release into another dye's detector, by deducting superfluous emission from either the FL1 or FL3 regions. Fluorescence color compensation was set using single-stained controls for each cell type, and fluorescence spillover was corrected (BD [21]). In some instances, the spillover could not be corrected without significantly affecting the counted gated region, and in these cases, compensation was not done manually. The fluorescence was collected as logarithmic signals.The scatter dot plots attained during flow cytometric analysis were separated into four quadrants, representing *Vibrio cholerae O1* cells with different physiological properties, and similar plots were obtained for *Vibrio cholerae O139* (data not shown). Each experimental sample was gated from the control population by scatter, and appropriate compensation was applied (Figures 2(a)–2(k)). Gating (R1) for all probes was organized on scatter plots of “forward scatter (FSC) versus side scatter (SSC)” to mimic the total bacterial population without any background particles/noise (Figure 2(a)), as described by Singh and Barnard [1]. In this study, data for two parameters were collected, and hence, a bivariate histogram (Figure 2) for gating was used, which divided the plot in four quadrants for the four possible combinations. Nebe-von Caron et al. [19] showed that when using pure bacterial cultures with flow cytometry, it is conceivable to “classify the bacteria by their light scatter signal” and that at considerably high bacterial numbers, interfering background particles and noise within the region can be excluded. In this study, intact cells (Figures 2(c) and 2(h)) were computed from FL1 and FL2 regions and the damaged cells (Figures 2(e) and 2(j)) from FL3 region. An intermediate subpopulation that presented membrane injury and is positive for both the CFDA-AM+PI and TO+PI probes has been categorized as being in the “injured” or “stressed” state of cells in other studies [1]. Stressed bacterial populations were computed from the FL1 vs. FL3 region (Figures 2(d), 2(f), 2(i), and 2(k)). The stained bacterial subpopulations were automatically counted from the number of events (bacterial cells) included inside the four quadrants with the BD CSampler™ software [1, 4]. Data was collected until 20000 events were acquired for every sample.

### 2.4. Data Analysis

Data was analysed using the BD C6 Accuri CSampler™ software and IBM SPSS statistics v26 software. The GraphPad Prism 8 software was used to illustrate graphs.  Figure 1 was drawn using BioRender imaging software. Simulated gastric fluid tests for each bacterial pathogen were done separately three times to screen for accuracy and reproducibility of the results. Data comparisons were performed, and significance was set at a 95% confidence interval (0.05).

## 3. Results

A double labelling assay was performed with flow cytometry with blends of probes: CFDA-AM, TO, and PI to distinguish between intact and stressed bacterial cells in samples comprising of live and dead bacterial cells (Figures 1 and 2). In the flow cytometry analysis as shown in Figure 2, *V. cholerae* bacterial cells were evaluated by light scatter, for esterase activity by the CFDA-AM probe and for membrane integrity with TO and PI probes simultaneously. This was determined by retaining or seepage of CFDA-AM, and TO and expelling or in-taking of PI. Bacterial cell events were distinguished by both esterase activity and membrane integrity. On the dot plots of red fluorescence vs. green fluorescence, four quadrants were arranged by crossline gating (Figure 2). Three mixed bacterial subpopulations were detected as follows: the intact/viable/vital subpopulation (green), stressed/injured/VBNC subpopulation (blue), and the damaged subpopulation (red) (Figure 2). Figures 2(b) and 2(g) are the unstained bacterial culture control showing no fluorescence. The remaining unstained (U) quadrant is representative of the background noise/debris/cells that have not taken up any dye and were disregarded in the data analysis.

**Figure 3:**
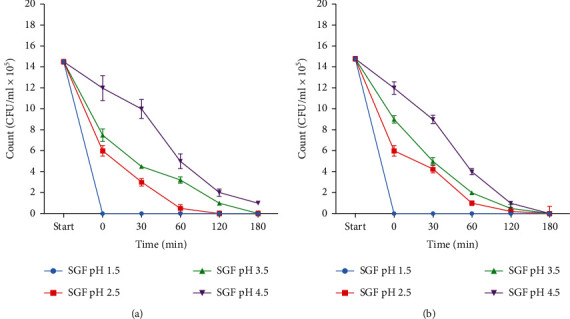
Culturable growth trends of *Vibrio cholerae* O1 (a) and *Vibrio cholerae* O139 (b) after exposure to simulated gastric fluid over 180 min. The experiments were repeated three times, and data represented as mean CFU/ml (SD).

In our previous study, after SGF acid exposure at each time interval, the bacterial cells were tested for their ability to grow as colonies on plate count agar [1], with the formation of colonies being a representation of culturable cells. In this study, the initial inoculum for *Vibrio cholerae* O1 and O139 was determined by plate count data and is mentioned in the Methods section. The inoculum was prepared at late stationary phase since bacterial cells are able to undergo radical physical adaptation to “fight” physical stresses [22]. Testing of SGF treatment at time 0 min occurred as soon as the bacterial cells were added to each flask of SGF pH. The bacterial inoculums decreased upon exposure to SGF for both *V. cholerae* O1 and O139 (Figure 3, it should be mentioned here that moderately similar results were obtained in our previous study). No pathogen was able to grow or form a colony on the agar at SGF pH 1.5 after initial acid exposure. Both *V. cholerae* had culturable growth in SGF from pH 3.5 to pH 4.5 with a decrease in population intensity as incubation increased (i.e., exposure to the SGF over 180 min). *V. cholerae* O1 was able to still form colonies at pH 4.5 for the duration of the acid treatment (180 min, Figure 3(b)). The culturable growth trend noted was a decrease in bacterial population over time, possibly due to the damaging effect of acid on bacterial cell membrane structure.

**Figure 4:**
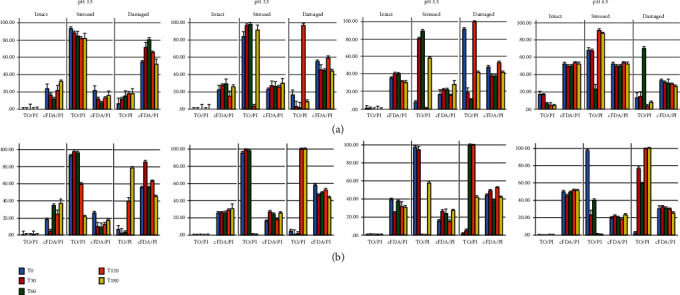
Percentage (mean *n* = 3) viability (TO/PI) and vitality (CFDA/PI) of (a) *Vibrio cholerae* O1 and (b) *Vibrio cholerae* O139 exposed to simulated gastric fluid at four pHs over a period of T0-T180 min. Bar graphs represent the physiological state detected at each time interval.

Figure 4 bar graphs represent the plots of all fluorescent-labelled bacterial particles at all simulated gastric fluid pH and at all sampling intervals. The bars show distinct different distributions for each bacterial cell type of intact, stressed, and damaged cells when using either a DNA probe (TO) or metabolic probe (CFDA-AM) for determining bacterial cell viability and activity, respectively. Cell count data (%) from FCM investigation were compared with the TO, CFDA-AM, and PI probes for the two *V. cholerae* populations exposed to varying pHs of SGF (Figure 4). The bacterial strains were grown in nutrient broth at a neutral (pH 7.01), and the starting inoculum size represented 100% of intact cells (data not shown, example can be seen in Figures 2(c) and 2(d)). As soon as the inoculum was added to the prewarmed SGF treatments at T0, it is presumed that initial acid shock occurred to the unadapted bacterial strains, which resulted in a predamaging effect on the membrane allowing for early penetration of the PI probe. The early entry of the PI probe resulted in the intact bacterial cell populations being relatively low for all four SGF pH (1.5, 2.5, 3.5, and 4.5) treatments at T0 min. Significant differences (*ρ* ≤ 0.05) in survivability (intact cell distribution) based on membrane integrity and activity were seen from 0 to 180 min for both *V. cholerae* O1 and *V. cholerae* O139 after exposure to SGF.

Although membrane integrity was compromised, metabolic esterase activity was evident (intact cells) as shown when labelling the cells with CFDA-AM for both strains and at all SGF pHs. The late stationary phase non-acid-adapted bacterial cells endured a partial loss of cell membrane integrity that allowed for the early penetration of TO and PI probes and shifted the cells into the stressed state from time 0 min at pH 1.5-pH 3.5 for *V. cholerae O1* and at all pHs for *V. cholerae O139.* From Figure 4, it can be seen that the CFDA-AM fluorescent probe was more effective in clarifying the vitality of bacterial cells for both strains compared to the TO fluorescent probe. *V. cholerae* O1 intact cells in SGF pH 1.5 was 0.24% (0 min) to 0.14% (180 min) and 23.68% (0 min) to 32.04% (180 min) when labelled with TO/PI and CFDA-AM/PI, respectively (Figure 4(a)). *V. cholerae* O139 intact cells in SGF pH 1.5 was 0.47% (0 min) to 0.16% (180 min) and 18.4% (0 min) 37.06% (180 min) when labelled with TO/PI and CFDA-AM/PI, respectively (Figure 4(b)). The phenomenon of the increase in the number of intact cells over time as shown by the CFDA-AM labelled cells can be explained by the “suicide stress response” as postulated by Dodd et al. [23] who said that “when bacteria are exposed to sublethal stresses, the growth of aerobically respiring bacterial cells in the log phase seizes; however, they retain metabolic function for an extended period of time.” The initial acid shock in this study and in our previous study [1] showed that at 0 min bacterial cell activity seized due to stress; however, over time (180 min) metabolic activity was restored. The CFDA-AM probe was able to detect a higher bacterial load in both the intact and stressed cell states at all SGF pH and time intervals tested to that of the TO probe. CFDA-AM probe staining is significantly different (*ρ* ≤ 0.05) to that of the TO probe. For both bacterial strains, the TO probe shifted 80-90% of all pH acid-exposed cells into the stressed and damaged state (0-60 min). Flow cytometry coupled with fluorescent probes was able to differentiate the status of gastric fluid stressed *Vibrio cholerae* based on composition, membrane integrity, and activity.

**Figure 5:**
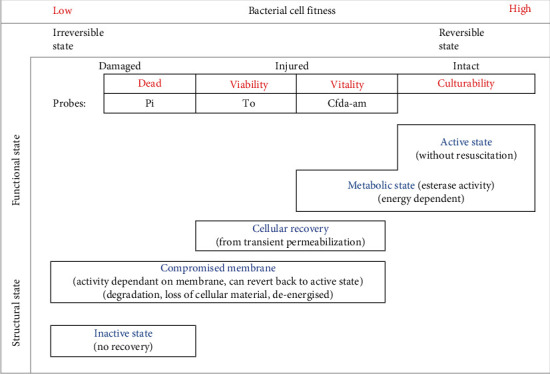
Classification of *Vibrio cholerae* cell fitness after acid stress and probe labelling in terms of functional and structural states.

Figure 5 shows the classification of bacterial cell fitness based on measurable cell characteristics, as determined in this study. Functional state (cellular components) and the structural state (membrane integrity) play a key role in establishing the fitness of bacteria after exposure to stressful circumstances. According to Strauber and Muller (2010), bacterial cell wall and cytoplasmic membranes “protect and stabilize a cell” against environmental stressors, with the membrane structure and function influencing the permeation of fluorescent probes to enter the cell. The peak form of fitness is a state of culturability (reproductive growth) that requires both metabolic activity and membrane integrity. In previous studies, we have shown that culturability can be lost during stressful conditions that may compromise the nucleic acid and structural potential of the cell [1]. This study showed that *Vibrio cholerae* can enter a “viable but nonculturable state” due to acid stress that causes membrane permeability [1]. This study further showed that *Vibrio cholerae* cell vitality and viability can be better distinguished by using fluorescent probes (CFDA-AM) for metabolic activity (Figure 5).

## 4. Discussion

The vitality and viability of *Vibrio cholerae* O1 and O139 after exposure to simulated gastric fluid (SGF) was assessed by applying fluorescent probes TO, CFDA-AM, and PI in blends with flow cytometry (FCM), by evaluating bacterial populations varying in relation of intact-stressed-damaged cells. The use of FCM to separate viable and nonviable bacteria after marking with probes “CFDA-Calcein AM: or PI has been shown for food microbiology, composting, seawater [8, 24–26], and for bacteria of the gastrointestinal tract [6, 27]. To our knowledge, this is the first study that used a combination of probes (DNA and enzymatic probes) to assess bacterial cellular status after exposure to a stressor-simulated gastric fluid. The fluorescence spectral measurements were obtained by using a portable and cost-effective flow cytometry system (BD Accuri C6™) that accurately quantified fluorescence signals in near real time. Fluorescence was measured from probes TO-, CFDA-AM-, and PI-stained *V. cholerae* samples after exposure to varying acidity levels of SGF over 180 min, and the percentage of viable (live), stressed (injured), and damaged (dead) cells present was predicted (Figures 2 and 4).

Thiazole orange, a permeant probe, can enter all cells irrespective of membrane integrity, binds to nucleic acids (DNA or RNA), and emits a green fluorescence signal. Propidium iodide, an impermeant nucleic acid probe, which only passes through compromised cell membranes, binds to DNA or RNA, and emits a red fluorescence signal [17]. CFDA-AM, a vital probe, is a cell permeable esterase substrate that is used for the assessment of viability [28]. The “acetoxymethyl ester” (AM) of the esterase substrate “5-carboxyfluorescein diacetate” (CFDA) allows this reagent to enter through cellular membranes, and when inside the bacterial cell, the lipophilic blocking and diacetate groups are cleaved by nonspecific esterase's, resulting in green fluorescence (Invitrogen: F34953). The permeant and impermeant probes can be combined to enable quick physical association of bacterial cells and total counting. Metabolic activity of stress-induced bacteria, in the absence of growth, plays an important role in establishing viability [17]. Bacterial pathogens may still cause disease when they are in an active but nonculturable status [29].

This study showed that membrane integrity grounded on viability staining with DNA-binding probes (TO), including PI, did considerably overestimate the stressed bacterial cell counts. This is due to injured cells being able to take up both probes and show a strong fluorescence of both the impermeant and permeant stains, since both PI and TO have a strong affinity to nucleic acid, thus over estimating the stressed cell count (Figure 4).

Staining with CFDA-AM + PI resulted in three cell populations (Figure 2) intact (CFDA-AM^+^), damaged (PI^+^), and stressed (CFDA-AM^+^, PI^+^). The stressed population detected by these probes is compromised (i.e., the cell membrane which has permeabilized due to SGF acid stress, but to a low extent). The stressed cells do not exclude PI anymore, and the membrane has “enough integrity” to maintain CFDA-AM in the cell. It is thought that PI has a stronger affinity for nucleic acid than CFDA-AM and can therefore displace CFDA-AM when considerable membrane damage is present, thus increasing red fluorescence (damaged cells) (Figure 4). However, it should be noted that the stressed “physiological status” is temporary and can progressively change towards cell damage or viability, and it is this “stressed” status in which cell death is not yet irreversible, and the bacteria may still recover [8].

Both *V. cholerae* strains were membrane stressed/damaged at all SGF pH (1.5, 2.5, 3.5, and 4.5) throughout 180 min (as shown by the TOPI probes, Figure 4); however, the strains did have limited growth (culturability) up to 120 min as pH (2.5, 3.5, and 4.5, Figure 3). Due to membrane integrity being a moderate indicator for viability [16], and culturability not necessarily showing the complete heterotrophic cell count [30], it was decided to include additional vitality test (CFDA-AM) to confirm the results. Because of the possibility of sublethal injury, it is important to use alternative methods to measure bacterial cell viability. The number of viable cells (based on vital staining by CFDA-AM) distinguished by FCM was continuously higher than the nucleic acid TO probe staining. This suggests that a large percentage of the *V. cholerae* O1 and O139 cells was enzymatically active after exposure to extreme gastric acid stress, i.e., they are able to hydrolyze CFDA-AM. FCM analysis of CFDA-AM-stained cells can therefore be used as a vitality indicator, being superior to the nucleic acid probe due to it shifting the acid exposed cells immediately. The PI probe is a good indicator for dead or damaged cells, due to it entering only permeabilized membranes.

This study showed the “value” of using different probes to provide single-cell physiological information on monitoring the heterogeneity of bacterial response to acid stress. It was deduced that if the loss of cellular activity is irreversible, bacterial cells can be deliberated to be dead and that an intact membrane is essential for conserving the ability of metabolic activity (Figure 5). Bacterial cells can recover from partial cell damage due to “stress” and revert back to an intact state, but if the membrane is permanently damaged, cell death will occur.

Lidstrom and Konopka [31] showed in their research that typical “growth in the response against stress across bacterial cells in culture does not exist, but there are intermediate states that depend on a threshold response mechanism, giving rise to physiologically distinct populations.” When bacterial pathogens are exposed to sublethal injury or stress, cellular changes will occur whereby morphological and functional components will be damaged or affected, such as cell wall, inner membrane, cytoplasmic membrane, numerous enzymes, and nucleic acids. It is also important to remember that not all cells within the bacterial population will undergo the same amount of injury and that varying stress factors will induce different types of injury/ies to the cell [3].

The detection of stress and damage to the cell membranes is indicative of the ability of the bacteria to be culturable. The results obtained from the culturability analysis (Figure 3) in this study and in our previous exploratory study only confirmed the incidence of live *Vibrio cholerae* after contact to SGF and did not take into account that these bacteria could still be “viable but nonculturable” (VBNC). VBNC bacteria remain metabolically active and cannot be cultured on media where they would normally form colonies [28, 32]. In this state, bacteria conserve metabolic activity, membrane integrity, respiration, and slow gene transcription, even though they have lost the ability to be cultured [33]. The SGF studies in our laboratory also showed that plate culturing cannot determine the intermediate survival states that bacteria may undergo after stress such as injury or the VBNC state [1]. In this study, no comparability was established between the culturability studies and flow cytometry analysis.

Rosenberg et al. [34] suggested in their study that membrane integrity and enzymatic activity, in a stressed setting, cannot determine the reproductive capability of cells, as this can only be measured by resuscitation with cultivation-based methods. The uncertainty of microbial plate culture-based methods to instantly provide the growth conditions, coupled with the inefficiency of measuring metabolic activity, and the likelihood of injured cells to recover, makes membrane integrity/vitality the ideal presumptive measurement for viability [35]. It can also be assumed from flow cytometry data that the stressed population of cells may have cellular metabolic activity, but due to the membrane damage, these cells cannot colonize on plate count agar. This occurrence can only be shown upon further investigation with cellular resuscitation and FCM bacterial cell sorting with plating on media for culturability. Since the acid shock of gastric fluid brought about injury/stress to the *Vibrio* cells, some of these “stressed” cells identified by FCM might have entered the VBNC state of survival. Future research will include the investigation of *Vibrio cholerae* extracellular vesicles (EVs), as these vesicles have been shown in other studies to be effective in bacterial defense mechanisms against stressors, quorum sensing, and bacterial “host-pathogen” relationships [36, 37]. EVs stimulate bacterial surface exchange and subsequent adaptation to the to the surrounding environment and can possibly show the mechanism survival with the dye uptake.

This study showed that although staining with the nucleic acid probe (TO) showed significantly lower viability, shifting most *Vibrio* cells into the stressed state (which is also an indication of the VBNC state), the vitality staining with the metabolic probe (CFDA-AM), and confirmed that these cells did still have activity in them (Figure 3). Flow cytometry coupled with fluorescent probes is therefore a more suitable method for the immediate and automatic detection of intact or compromised bacteria in any environment, because it can provide vitality and viability bacterial data in real time at the cellular level. This method can be applied to study bacterial cell adaptations and survival to environmental factors, antibiotics, and human intestinal tracks to better understand how these cells survive and overcome challenges to cause human infection and continue the spread of disease. This immediate description of a bacterial population will play a critical role in quality control programs, especially during diagnosis, to prevent illness.

## 5. Conclusion

Flow cytometry is generally considered the most appropriate tool when it comes to the determination of bacterial “physiological states.” It is a “valuable method” for the identification of stressed bacteria in applications, as these bacteria may be in a nonculturable but still active state. In response to environmental stresses (such as pH variation), bacteria may enter into a viable but nonculturable (VBNC) state and later resuscitate when favorable conditions return [38]. Traditional plate counts can miscalculate this risk, but flow cytometry would be able to detect all cells and distinguish the active but nonculturable state. Due to the surreptitious nature of VBNC cells, their role in biological systems cannot be underestimated. The method used in this study, supported with the data gathered, can assist in determining the possible risk linked to consuming bacterial-tainted foods, water, or processing environments where pH is involved, taking bacterial cell damage into deliberation. This study showed that FCM offers real measurements for each acid-stressed bacterial cell and in less time. With an appropriate combination of probes, the damage caused by SGF can be measured and the number of affected cells determined, and additionally, using fluorescent probes, metabolic activity can also be determined. A combination of CFDA-AM and PI fluorescent probe labelling can be used to study the vitality and population dynamics of (stressed) mixed cultures of bacteria. Combining these assays with FCM enables fast measurements of the physiological status of stressed bacteria and of subpopulations of individual cells in research and other biotechnological applications.

## Data Availability

Additional datasets used during the current study are available from the corresponding author on reasonable request.

## Supplementary Materials

Supplementary Materials The flow cytometry data used to support the findings of this study for each bacterial strain exposed to SGF and subsequent probe staining is included within the supplementary data file.
