# Supplementary material for: A Possible Flow Cytometry-Based Viability and Vitality Assessment Protocol for Pathogenic Vibrio cholerae O1 and O139 Postexposure to Simulated Gastric Fluid
Source: Biomed Res Int. 2021 Jun 8;2021:5551845. doi: 10.1155/2021/5551845 (PMC8208853; doi:10.1155/2021/5551845)
Supplement: Supplementary Materials — The flow cytometry data used to support the findings of this study for each bacterial strain exposed to SGF and subsequent probe staining is included within the supplementary data file. [file 5551845.f1.pdf]

SAMPLE	STAIN	BACTERIA	pH	STATE	D1T0	D2T0	D3T0	D1T30	D2T30	D3T30	D1T60	D2T60	D3T60	D1T120	D2T120	D3T120	D1T180	D2T180	D3T180
1	TO+PI	V. cholerae 01	1.5	Intact	0,33	0,17	0,22	0,07	0,00	0,01	0,01	0,00	0,00	0,12	0,04	0,12	0,19	0,11	0,11
2	TO+PI	V. cholerae 01	1.5	Stressed	91,97	94,99	92,27	87,30	88,63	88,42	84,23	85,02	84,41	81,67	85,27	78,15	83,15	81,19	81,45
3	TO+PI	V. cholerae 01	1.5	Damaged	7,70	4,84	7,51	12,63	11,37	11,57	15,77	14,99	15,58	18,20	14,70	21,73	16,65	18,69	18,45
4	TO+PI	V. cholerae 01	2.5	Intact	0,08	0,02	0,03	0,07	0,02	0,02	0,07	0,02	0,03	0,00	0,00	0,00	0,02	0,00	0,00
5	TO+PI	V. cholerae 01	2.5	Stressed	84,56	84,31	82,53	96,23	97,24	96,01	96,52	99,00	98,14	7,03	2,57	1,39	91,80	91,15	90,66
6	TO+PI	V. cholerae 01	2.5	Damaged	17,37	15,67	17,44	3,69	2,75	3,97	3,41	0,99	1,83	92,98	97,43	98,62	8,19	8,85	9,34
7	TO+PI	V. cholerae 01	3.5	Intact	0,99	0,79	0,84	0,55	0,41	0,34	0,01	0,01	0,01	0,01	0,01	0,00	0,00	0,01	0,00
8	TO+PI	V. cholerae 01	3.5	Stressed	7,60	7,60	8,13	83,57	79,43	79,27	90,84	87,85	88,88	0,01	0,00	0,01	56,84	57,44	59,73
9	TO+PI	V. cholerae 01	3.5	Damaged	91,41	91,61	91,04	15,87	20,15	20,38	9,15	12,15	11,12	99,99	100,00	99,99	43,16	42,55	40,27
10	TO+PI	V. cholerae 01	4.5	Intact	17,81	17,29	17,03	17,97	16,15	17,48	6,47	6,73	6,06	5,10	4,82	4,71	4,14	4,19	4,29
11	TO+PI	V. cholerae 01	4.5	Stressed	67,07	69,43	69,13	66,92	67,61	68,61	22,19	23,36	23,75	90,96	91,23	92,05	85,83	88,29	88,95
12	TO+PI	V. cholerae 01	4.5	Damaged	13,08	13,28	13,83	15,12	16,24	13,90	71,34	69,91	70,19	3,94	3,95	3,23	10,04	7,52	6,76
13	CFDA+PI	V. cholerae 01	1.5	Intact	24,11	23,50	23,42	16,86	15,82	16,58	13,08	11,53	11,20	21,87	21,20	21,90	31,65	32,59	32,00
14	CFDA+PI	V. cholerae 01	1.5	Stressed	22,31	21,65	21,24	11,87	12,36	11,43	9,50	6,85	6,73	12,58	12,79	12,46	16,40	15,52	15,61
15	CFDA+PI	V. cholerae 01	1.5	Damaged	53,58	54,85	55,32	71,27	71,82	72,02	77,45	81,62	82,07	65,55	65,98	65,61	51,95	51,89	52,39
16	CFDA+PI	V. cholerae 01	2.5	Intact	20,39	22,50	24,32	26,24	27,83	28,36	28,48	29,90	28,92	14,37	15,26	16,26	25,44	25,11	27,04
17	CFDA+PI	V. cholerae 01	2.5	Stressed	30,79	29,10	8,49	27,47	26,20	27,34	25,95	25,70	26,86	37,13	37,33	2,58	29,74	30,81	29,48
18	CFDA+PI	V. cholerae 01	2.5	Damaged	48,81	48,41	67,19	46,31	45,99	44,30	45,57	44,42	44,22	48,52	47,41	81,15	44,82	44,08	43,48
19	CFDA+PI	V. cholerae 01	3.5	Intact	35,94	32,97	37,14	41,02	39,70	40,90	38,46	41,53	40,65	32,11	29,33	32,66	28,85	30,74	30,94
20	CFDA+PI	V. cholerae 01	3.5	Stressed	12,31	25,28	12,16	22,05	22,12	21,52	23,32	21,12	21,72	10,84	25,54	10,74	28,83	27,49	27,20
21	CFDA+PI	V. cholerae 01	3.5	Damaged	51,75	41,77	50,72	36,93	38,18	37,60	38,24	37,35	37,63	57,05	45,13	56,60	42,32	41,77	41,87
22	CFDA+PI	V. cholerae 01	4.5	Intact	55,23	50,82	51,29	48,59	50,45	50,43	50,38	50,10	50,17	58,78	51,77	50,62	52,78	52,65	51,57
23	CFDA+PI	V. cholerae 01	4.5	Stressed	8,09	18,04	17,27	19,79	18,99	18,88	19,16	20,18	19,49	10,03	20,41	21,28	20,37	20,91	21,84
24	CFDA+PI	V. cholerae 01	4.5	Damaged	36,68	31,16	31,44	31,62	30,55	30,67	30,46	29,72	30,34	31,17	27,80	28,10	26,83	26,44	26,61
25	TO+PI	V. cholerae 0139	1.5	Intact	0,72	0,39	0,29	0,11	0,11	0,09	0,44	0,23	0,23	0,89	0,89	0,77	0,17	0,14	0,15
26	TO+PI	V. cholerae 0139	1.5	Stressed	92,60	93,17	92,98	97,73	97,44	97,37	96,94	96,22	96,32	58,45	61,99	58,30	21,06	21,25	21,59
27	TO+PI	V. cholerae 0139	1.5	Damaged	6,68	6,45	6,72	2,16	2,45	2,53	2,62	3,55	3,43	40,66	37,12	40,94	78,75	78,60	78,26
28	TO+PI	V. cholerae 0139	2.5	Intact	0,34	0,11	0,06	0,06	0,05	0,04	0,06	0,05	0,03	0,00	0,01	0,00	0,00	0,01	0,01
29	TO+PI	V. cholerae 0139	2.5	Stressed	95,20	95,68	95,14	99,00	99,00	98,96	98,15	97,99	97,70	0,02	0,03	0,05	0,03	0,03	0,01
30	TO+PI	V. cholerae 0139	2.5	Damaged	4,46	4,21	4,81	0,95	0,95	1,00	1,79	1,96	2,26	99,98	99,96	99,96	99,97	99,97	99,98
31	TO+PI	V. cholerae 0139	3.5	Intact	0,57	0,42	0,30	0,02	0,02	0,02	0,01	0,00	0,01	0,00	0,00	0,01	0,00	0,01	0,00
32	TO+PI	V. cholerae 0139	3.5	Stressed	97,53	97,72	98,13	94,68	94,77	95,18	0,03	0,04	0,01	0,02	0,03	0,02	56,84	57,44	59,73
33	TO+PI	V. cholerae 0139	3.5	Damaged	1,90	1,87	1,57	5,30	5,21	4,80	99,96	99,96	99,98	99,98	99,97	99,98	43,16	42,55	40,27
34	TO+PI	V. cholerae 0139	4.5	Intact	0,06	0,07	0,05	0,09	0,18	0,09	0,15	0,15	0,11	0,06	0,09	0,06	0,04	0,06	0,03
35	TO+PI	V. cholerae 0139	4.5	Stressed	97,76	96,66	96,96	24,82	21,42	23,53	40,77	41,32	39,22	0,73	0,68	0,73	0,14	0,17	0,18
36	TO+PI	V. cholerae 0139	4.5	Damaged	2,18	3,27	2,99	75,09	78,40	76,37	59,08	58,52	60,66	99,22	99,23	99,21	99,81	99,77	99,79
37	CFDA+PI	V. cholerae 0139	1.5	Intact	18,30	18,62	18,30	4,01	4,63	4,75	34,99	34,28	34,71	24,47	24,23	23,93	36,42	36,99	37,76
38	CFDA+PI	V. cholerae 0139	1.5	Stressed	26,38	26,22	25,08	11,23	8,94	9,47	10,06	8,66	9,36	12,88	12,41	12,20	17,13	18,17	17,67
39	CFDA+PI	V. cholerae 0139	1.5	Damaged	55,33	55,18	56,61	84,77	86,47	85,78	54,96	57,10	55,97	62,65	63,36	63,87	46,45	44,84	44,53
40	CFDA+PI	V. cholerae 0139	2.5	Intact	23,84	26,04	27,85	26,54	26,95	25,65	26,98	25,81	26,21	28,19	30,38	29,90	30,30	31,30	30,96
41	CFDA+PI	V. cholerae 0139	2.5	Stressed	29,45	7,81	12,90	27,30	26,92	26,96	25,07	25,17	23,64	35,84	9,71	9,50	25,75	26,15	25,00
42	CFDA+PI	V. cholerae 0139	2.5	Damaged	46,71	66,15	59,24	46,16	46,13	47,40	47,95	49,02	50,15	35,95	59,91	60,60	43,95	42,57	44,06
43	CFDA+PI	V. cholerae 0139	3.5	Intact	36,57	41,11	40,98	23,72	24,63	24,62	38,52	37,31	36,86	27,01	34,05	33,96	29,04	32,03	31,71
44	CFDA+PI	V. cholerae 0139	3.5	Stressed	23,47	12,19	12,61	26,91	26,04	26,65	24,06	24,09	24,20	26,19	9,83	10,22	28,02	26,47	26,62
45	CFDA+PI	V. cholerae 0139	3.5	Damaged	39,97	46,69	46,41	49,37	49,33	48,73	37,41	38,60	38,93	46,80	56,10	55,84	42,96	41,48	41,67
46	CFDA+PI	V. cholerae 0139	4.5	Intact	48,83	51,07	50,03	42,70	45,85	47,11	48,27	49,89	48,54	49,38	52,40	53,27	51,13	50,81	51,67
47	CFDA+PI	V. cholerae 0139	4.5	Stressed	20,80	19,09	18,88	22,83	22,24	21,05	20,69	18,78	20,30	19,33	17,63	17,52	23,63	23,74	22,72
48	CFDA+PI	V. cholerae 0139	4.5	Damaged	30,39	29,84	31,11	34,45	31,89	31,86	31,04	31,33	31,16	31,29	29,97	29,23	25,24	25,45	25,61
